# Proteases Production and Chitin Preparation from the Liquid Fermentation of Chitinous Fishery By-Products by *Paenibacillus elgii*

**DOI:** 10.3390/md19090477

**Published:** 2021-08-25

**Authors:** Dan-Hsin Lee, Chien Thang Doan, Thi Ngoc Tran, Van Bon Nguyen, Anh Dzung Nguyen, Chuan-Lu Wang, San-Lang Wang

**Affiliations:** 1 Department of Chemistry, Tamkang University, New Taipei City 25137, Taiwan; ivylee615@gmail.com (D.-H.L.); dcthang@ttn.edu.vn (C.T.D.); 2 Faculty of Natural Sciences and Technology, Tay Nguyen University, Buon Ma Thuot 630000, Vietnam; ttngoc@ttn.edu.vn; 3 Doctoral Program in Applied Sciences, Tamkang University, New Taipei City 25137, Taiwan; 4 Institute of Biotechnology and Environment, Tay Nguyen University, Buon Ma Thuot 630000, Vietnam; nvbon@ttn.edu.vn (V.B.N.); nadzung@ttn.edu.vn (A.D.N.); 5 Department of Fashion Beauty Design, Lan Yang Institute of Technology, Yilan County 26141, Taiwan; wang_chuan_lu@gmail.com; 6 Life Science Development Center, Tamkang University, New Taipei City 25137, Taiwan

**Keywords:** chitin, deproteinization, dye adsorption, *Paenibacillus elgii*, protease

## Abstract

Chitinous fishery by-products have great application in the production of various bioactive compounds. In this study, *Paenibacillus elgii* TKU051, a protease-producing bacterial strain, was isolated using a medium containing 1% squid pens powder (SPP) as the sole carbon/nitrogen (C/N) source. *P. elgii* TKU051 was found to produce at least four proteases with molecular weights of 100 kDa, 57 kDa, 43 kDa, and 34 kDa (determined by the gelatin zymography method). A *P. elgii* TkU051 crude enzyme cocktail was optimally active at pH 6–7 and 60 °C. The 2,2-diphenyl-1-picrylhydrazyl radical scavenging activity and α-glucosidase inhibitory activity of the hydrolysates obtained from the hydrolysis of shrimp shell powder, shrimp head powder, shrimp meat powder, fish head powder and soya bean powder catalyzed by the *P. elgii* TkU051 crude enzyme cocktail were also evaluated. *P. elgii* TKU051 exhibited a high deproteinization capacity (over 94%) on different kinds of shrimp waste (shrimp heads and shells; fresh and cooked shrimp waste; shrimp waste dried by oven and lyophilizer), and the Fourier-transform infrared spectroscopy profile of the chitin obtained from the deproteinization process displayed the characteristic of chitin. Finally, the obtained chitin exhibited an effect comparable to commercial chitin in terms of adsorption against Congo Red (90.48% and 90.91%, respectively). Thus, *P. elgii* TKU051 showed potential in the reclamation of chitinous fishery by-products for proteases production and chitin extraction.

## 1. Introduction

Chitin is a natural biological polymer present in the exoskeleton of crustaceans such as shrimps, crabs, and cephalopods, the pen of squids and the cell wall of fungi. The abundance of chitin in nature is second only to cellulose [1] and is mainly composed of *N*-acetyl-D-glucosamine with β-(1,4) linkage. According to reports, the annual production of shrimp and crab shell waste is 6–8 million tons globally [2]. The waste of shrimp and crab shells containing chitin is one of the main sources of chitin extraction. The industrial extraction of chitin from shrimp and crab shells is broadly done in two steps: deproteinization with NaOH and demineralization with HCl. However, the use of chemical methods to extract chitin causes a high level of pollution. Therefore, the use of microbial fermentation to produce protease to remove protein is a potentially environmentally friendly method [3,4,5].

Proteases are a group of enzymes that can hydrolyze peptide bonds of proteins. They participate in many biological processes involving synthesis and regulation. Proteases are also important industrial enzymes that account for over 60% of the total sales of industrial enzymes [6]. Among protease-producing sources, proteases derived from microorganisms have great potential due to the short growth cycle of the microbes and ease of separation. Besides being commonly used in the food industry to modify protein-rich materials, proteases are also widely used in the leather processing and washing industries [4]. In this study, protease was used to deproteinize seafood processing by-products for the extraction of chitin.

*Paenibacillus* was originally classified as *Bacillus* until 1993, and was later revised by Shida et al. in 1997 [7,8,9]. *Paenibacillus elgii* was formally proposed as a new species by Kim et al. in 2004 [9]. Like most *Paenibacillus* species, *P. elgii* has good antibacterial and antifungal abilities, so it is mostly used for microbial control applications. In addition to synthesizing antibiotics, *P. elgii* can also produce chitinase and protease through fermentation. Chitin is one of the primary components of fungal cell walls. The fungal cell wall can be hydrolyzed by chitinase to achieve a good antibacterial effect [10,11]. Protease can also hydrolyze proteins to produce peptides with antioxidant or antibacterial effects, exhibiting good antibacterial properties [12]. However, studies on the production and the application of *P. elgii* proteases for the utilization of marine processing by-products are limited.

With the rapid industrial development, wastewater discharged from factories has become one of the main sources of water pollution. Currently, more than 9000 kinds of dyes have been put into use in factories. According to their solubility and chemical properties, these dyes can be categorized as acid dyes, basic dyes, direct dyes, mordants, vat dyes, reactive dyes, disperse dyes, azo dyes, and sulfur dyes. Because of the low biodegradability of dyes, the traditional biological treatment process of sewage treatment plants cannot effectively treat dye wastewater, making the removal of water-soluble dyes very challenging [13,14]. Currently, there are two main methods to remove dyes: (1) transfer dye molecules to solid or activated sludge, and (2) completely destroy dye molecules [15,16]. Among the dye adsorbing agents, chitin has been wildly reported that have an excellent dye adsorption effect [17,18,19]. 

In this study, *P. elgii* TKU051, a protease-producing bacterial strain, was isolated from the soils of Tamkang University using a medium containing squid pens (commonly for isolating *Paenibacillus* strains) [20,21,22]. The production of proteases by this strain was analyzed by the gelatin zymography method. To determine the potential use of *P. elgii* TKU051 proteases, its crude enzyme cocktail was used to catalyze the hydrolysis reaction of some proteinaceous materials, and the obtained hydrolysates were used to evaluate antioxidant and α-glucosidase inhibitory activities. There are only a few reports on the application of *Paenibacillus* strains for deproteinization of chitinous material; thus, this study also suggests that *P. elgii* TKU051 can be used for the deproteinization of different kinds of shrimp waste.

## 2. Results and Discussions

### 2.1. Screening a Protease-Producing Bacterial Strain and the Production of Extracellular Proteases by P. elgii TKU051 

The bacterial strains were isolated from the soils of Tamkang University (New Taipei City, Taiwan) using 1% squid pen powder (SPP) as the sole C/N source [4]. In the protease assay, strain TKU051 showed the highest activity and was selected for further assessments. To determine the phylogenetic position of strain TKU051, sequencing of its 16s rRNA and homology analysis was performed. Based on the result, strain TKU051 exhibited the highest similarity (around 95%) with two species of the genus *Paenibacillus*, *P. elgii*, and *P. tyrfis*. The strain TKU051 was further taxonomically characterized based on morphological, physiological, and biochemical characteristics [23]. According to the results, TKU051 is a Gram-variable, rod-shaped, motile, and facultative anaerobic bacterium. This strain can produce catalase, α-glucosidase, β-glucosidase, protease, but cannot produce oxidase, and urease. It is also negative for L-arabinose and D-xylose utilization tests. These results cumulatively indicate that strain TKU051 is a member of species *P. elgii*. In a previous report, *P. elgii* was described for its ability to promote plant growth, inhibit the growth of pathogenic fungi and bacteria, produce extracellular chitinase, and resist the root-knot nematode [24]. This work, therefore, would further expand the ability of *P. elgii* for protease production and chitin preparation using chitinous fishery by-products as the unique carbon and nitrogen source.

**Figure 1:**
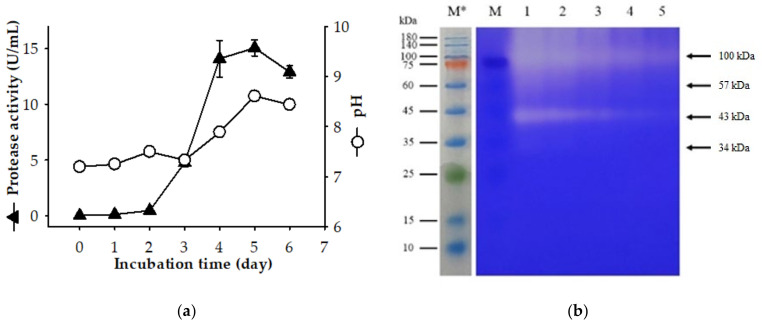
Time courses analysis of protease production by *P. elgii* TKU051 on SPP-containing medium (**a**) and gelatin zymography profile of the culture supernatant (**b**). The error bars are the standard deviation of three replications. M, protein markers; 1–5, 2-fold, 4-fold, 8-fold, 16-fold, and 32-fold diluted supernatant. * The gel before being stained by Coomassie brilliant blue solution.

The production of extracellular protease by *P. elgii* TKU051 on the SPP-containing medium is shown in Figure 1a. The maximal protease production was observed to be 14.10 U/mL on day 4, 15.07 U/mL on day five, and 12.91 U/mL on day six. The secretome of *P. elgii* TKU051 over SPP-containing medium was examined by SDS-PAGE containing 0.05% gelatin to identify the protease expression. As shown in Figure 1b, *P. elgii* TKU051 secreted at least four proteases of molecular weights (MWs) of 100 kDa, 57 kDa, 43 kDa, and 34 kDa. Of these, the 100 kDa and 43 kDa proteases were observed to be the major contributors of the proteolytic activity of the supernatant when these enzymes produced gelatin hydrolysis bands stronger than those produced by 57 kDa and 34 kDa proteases. The summary of the MW of the proteases generated by the *Paenibacillus* genus is shown in Table 1. Similar to *P. elgii* TKU051, several *Paenibacillus* strains such as *P. larvae, P. polymyxa* SCE2, and *P. peoriae* NRRL BD-62 were found to secrete four proteases into the culture medium [25,26]. However, the MW profiles of the proteases produced by *P. elgii* TKU052 were different from those produced by the strains listed above. There are only a few reports of protease produced by *P. elgii*. Kim et al. (2019) reported that *P. elgii* HOA73 could produce a protease, although its MW was not determined [27]. Thus, this result is a significant contribution to the knowledge of proteases of the *Paenibacillus* genus.

### 2.2. Biochemical Characterization of P. elgii TKU051 Crude Enzyme Cocktail

#### 2.2.1. Effect of Temperature and pH

**Figure 2:**
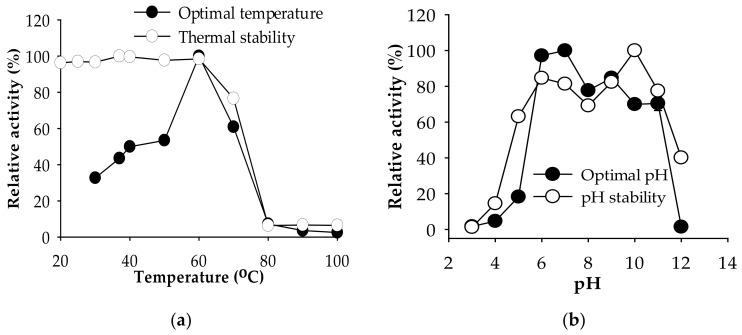
Effect of temperature (**a**) and pH (**b**) on the activity of *P. elgii* TKU051crude enzyme cocktail. The error bars are the standard deviation of three replicates of the assay.

The effect of temperature on the crude enzyme cocktail of *P. elgii* TKU051 is graphically presented in Figure 2. The protease activity was the highest at 60 °C and drastically decreased at higher temperatures, indicating that the optimal temperature of the crude enzyme cocktail is 60 °C. Likewise, several proteases from *Paenibacillus* have an optimum temperature at 60 °C, such as *P. mucilaginosus* TKU032 [19] and *Paenibacillus* sp. TKU052 [21]. The crude enzyme cocktail was thermally stable up to 60 °C and retained over 70% of its original activity at 70 °C, indicating its high thermo-stability. The optimal pH of the *P. elgii* TKU051 crude enzyme cocktail was determined in different buffer solutions (pH 3–12). The optimal pH was found to be 6–7 and it exhibited a high level of activity (over 70% of relative activity) from pH 6 to 11. In accordance with results similar to some alkaline proteases in previous studies [4,34], it can be speculated that the *P. elgii* TKU051 crude enzyme cocktail contains alkaline protease. The wide optimal pH range and pH stability of the *P. elgii* TKU051 crude enzyme cocktail may provide it with a greater ability to act at various pH conditions.

#### 2.2.2. Effect of Metal Ions, Inhibitors, Surfactants, and Reducing Agents

The effects of metal ions, inhibitors, surfactants, and reducing agents on the protease activity of *P. elgii* TKU051 crude enzyme cocktail are listed in Table 2. Among them, Ba^2+^ showed no significant effect, while Fe^2+^ slightly inhibited the protease activity (retained 87.24% of the activity). Cu^2+^ and Zn^2+^ could completely inhibit the enzyme activity whereas Mg^2+^ and Ca^2+^ could slightly enhance (113.34 and 108.12%, respectively) the protease activity of *P. elgii* TKU051 crude enzyme cocktail. Notably, Mn^2+^ could strongly enhance (364.04%) the protease activity of *P. elgii* TKU051 crude enzyme cocktail. The enzymes also showed a high tolerance to nonionic surfactants (Tween 20, Tween 40, and Triton X-100) and could even increase the protease activity of the *P. elgii* TKU051 crude enzyme cocktail (177.26, 204.59 and 127.96%, respectively). However, strong ionic surfactants such as SDS strongly inhibited the enzyme activity at 2 mM (22.50%). A reducing agent such as *β*-ME (*β*-mercaptoethanol) also partially inhibited protease activity at a concentration of 2 mM (88.91%). EDTA (ethylenediaminetetraacetic acid) and PMSF (phenylmethylsulfonyl fluoride), a metalloprotease inhibitor and a serine protease inhibitor, respectively, could effectively inhibit the protease activity (20.16 and 52.81%, respectively). Thus, these results indicate that the *P. elgii* TKU051 crude enzyme cocktail may contain metalloprotease and serine protease. Other metalloproteases and serine proteases produced by *Paenibacillus* have been reported [19,22,28].

#### 2.2.3. Substrate Specificity

The protease activity of the *P. elgii* TKU051 crude enzyme cocktail on various substrates was examined and the results are summarized in Table 3. The *P. elgii* TKU051 crude enzyme cocktail displayed activity in the order of casein > keratin > hemoglobin > albumin > myoglobin > fibrin > elastin > gelatin. A higher activity (58.85%) on keratin indicated the potential of *P. elgii* TKU051 crude enzyme cocktail to be used in the leather industry for de-hairing. Some proteinaceous materials such as shrimp shell powder (SSP), shrimp head powder (SHP), shrimp meat powder (SMP), fish head powder (FHP), and soya bean powder (SBP) were also used as the substrate for the *P. elgii* TKU051 crude enzyme cocktail to examine its protease activity on other substrates. *P. elgii* TKU051 crude enzyme cocktail expressed 28.49–44.50% of the protease activity on these materials compared to a casein substrate. This indicates that *P. elgii* TKU051 crude enzyme cocktail may be employed to obtain hydrolysates from SSP, SHP, SMP, FHP, and SBP.

### 2.3. Evaluation of the 2,2-Diphenyl-1-Picrylhydrazyl (DPPH) Radical Scavenging Activity and α-Glucosidase Inhibitory Activity of Some Proteinaceous Material Hydrolysates

SSP, SHP, SMP, FHP, and SBP were hydrolyzed by *P. elgii* TKU051 crude enzyme cocktail for 4 h to obtain hydrolysate solutions. The mixture of the proteinaceous materials and the enzyme cocktail at 0 h was also used as the control group. The hydrolysis process was eliminated by heating the mixture of enzyme and material at 100 °C for 10 min. 

**Figure 3:**
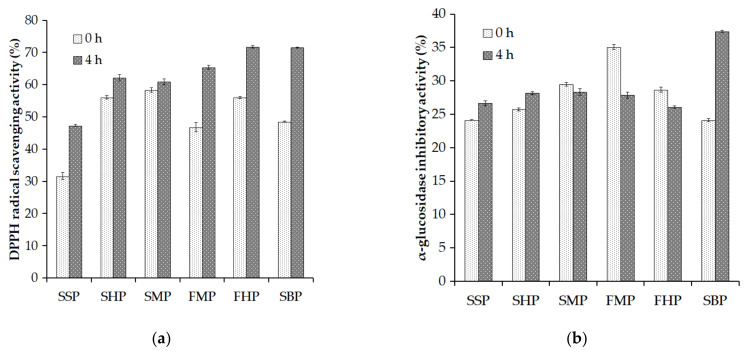
Evaluation of the 2,2-diphenyl-1-picrylhydrazyl (DPPH) radical scavenging activity (**a**), and α-glucosidase inhibitory activity (**b**) of some proteinaceous material hydrolysates. The error bars are the standard deviation of three replications.

Antioxidant-rich foods can support the human body against the negative impacts of free radicals. Among them, bioactive peptides are potent antioxidants used in food and medicines [35]. The antioxidant property of peptides is related to their radical scavenging capacity [36]. Thus, DPPH radical scavenging activity assay was used to explore the antioxidant activity of the hydrolysates from SSP, SHP, SMP, FHP, and SBP. As shown in Figure 3a, all the hydrolysate solutions exhibited a higher DPPH radical scavenging activity than their initial materials. This indicates that the activity of *P. elgii* TKU051 crude enzyme cocktail on the material could release antioxidant peptides into the solution. Among them, hydrolysates of FHP and SBP exhibited better antioxidant activity than those of others, and also exhibited over 70% of the DPPH radical scavenging activity.

The α-glucosidase inhibitor may regulate blood glucose levels and, as a result, prevent the onset of diabetes [37]. In this study, the hydrolysates of SSP, SHP, SMP, FHP, and SBP were used to explore α-glucosidase inhibitory activity, and the results are shown in Figure 3b. All the hydrolysates and their starting materials exhibited α-glucosidase inhibitory activity in a range of 24.11–37.39%. The hydrolysates of SPP, SHP and SBP exhibited higher α-glucosidase inhibitory activity than their initial materials, while the hydrolysates from SMP, FMP, and FHP showed lower results. The difference in the α-glucosidase inhibitory activity among the hydrolysates and starting materials may relate to their amino acid compositions and sizes [38]. In this study, the SBP hydrolysate exhibited the highest α-glucosidase inhibitory activity (37.39%). Thus, these results suggest that SBP hydrolysates produced by *P. elgii* TKU05s crude enzyme cocktail can potentially become antioxidant and α-glucosidase inhibitor candidates.

### 2.4. Deproteinization Ability of P. elgii TKU051

#### 2.4.1. Type of Shrimp Waste

**Figure 4:**
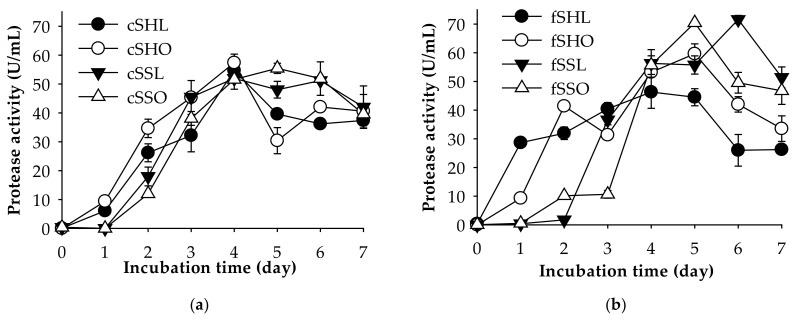
Protease production of *P. elgii* TKU051 on media containing different kinds of cooked shrimp wastes (**a**) and fresh shrimp wastes (**b**). The error bars are the standard deviation of three replicates.

One of the applications of protease-producing bacterial strains is in the deproteinization step of chitin preparation. Further investigation indicated that *P. elgii* TKU051 could produce chitinase (a maximal chitinase activity of 0.74 U/mL on day five of the fermentation period) on SPP-containing medium, whereas insignificant chitinolytic activity was observed when culturing this strain on shrimp head powder-containing medium and shrimp shells powder-containing medium. Thus, *P. elgii* TKU051 could be used to remove protein from shrimp waste, such as the heads and the shells. Likewise, shrimp waste is often used as sources of chitin extraction due to their large yield and low price. For deproteinization using *P. elgii* TKU051, fresh and cooked shrimp wastes (shells and heads) with different drying processes were chosen. They included fresh shrimp heads dried in an oven (fSHO), fresh shrimp heads dried by a lyophilizer (fSHL), fresh shrimp shells dried in an oven (fSSO), fresh shrimp shells dried by a lyophilizer (fSSL), cooked shrimp heads dried in an oven (cSHO), cooked shrimp heads dried by a lyophilizer (cSHL), cooked shrimp shells dried in an oven (cSSO), and cooked shrimp shells dried by a lyophilizer (cSSL) The results are shown in Table 4. The deproteinization rate of all types of shrimp waste reached about 94.36–96.86% after seven days of fermentation, and the residual protein of the shrimp wastes after fermentation was about 1.51–2.68%. Compared to other reports, shrimp waste showed a broad range of deproteinization rate, such as 95% (*Brevibacillus parabrevis* TKU046) [4], 97.9% (*Pediococcus acidilactici* CFR2182) [39], 84% (*B. subtilis*) [40], 40–72% (*Pseudomonas aeruginosa* K-187) [3], and 45% (*P. mucilaginosus* TKU032) [19]. These results indicate that using *P. elgii* TKU051 fermented shrimp waste has a high potential to remove protein, and the kinds of shrimp waste may not significantly affect the protein removal capability. The protease productivity of *P. elgii* TKU051 on different kinds of shrimp waste is shown in Figure 4. Among the shrimp waste, *P. elgii* TKU051 expressed higher protease activity on fSSO (70 U/mL, on day five) and fSSL (70 U/mL on day six). Additionally, the cooking process may affect the protease expression of *P. elgii* TKU051 when the enzyme production on fresh shrimp shells was higher than cooked ones (Figure 4a,b). By using an oven, the need for equipment is reduced compared to using a lyophilizer, and a large number of samples can be processed at one time. Thus, fSSO was selected as the material for further assays.

#### 2.4.2. Amount of Shrimp Waste

**Figure 5:**
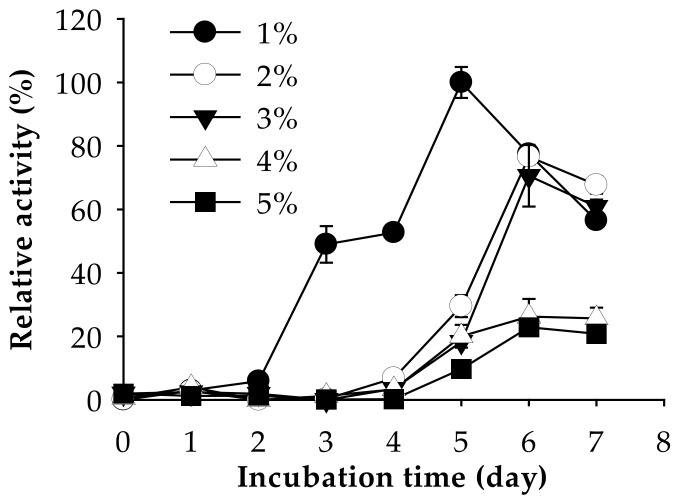
Effect of fSSO concentration on protease activity of *P. elgii* TKU051. The error bars are the standard deviation of three replicates.

The effect of fSSO amount (1–5%, *w/v*) on the deproteinization rate was also tested. Among them, only 1% fSSO (*w/v*) maintained a high deproteinization rate (97.45 %) after seven days of fermentation (Table 5). At higher fSSO amounts (2–5%), a lower deproteinization rate was observed (33.34–50.60%). Likewise, the highest protease activity was observed at 1% fSSO (Figure 5). The residual protein in 1% fSSO after fermenting was calculated to be only at 1.11%, whereas that in 2–5% fSSO was 21.51–29.08%. This result indicates that 1% fSSO was the most suitable amount for deproteinization and protease production by *P. elgii* TKU051.

### 2.5. Chitin Extraction after Liquid Fermentation 

**Figure 6:**
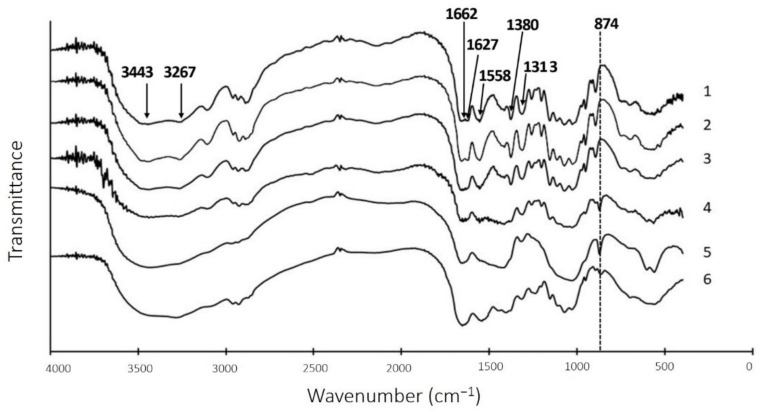
FT-IR profile of chitins prepared from fSSO by fermentation and alkaline deproteinization. **1**, commercial chitin; **2**, chitin prepared using alkaline deproteinization; **3**: chitin prepared using fermentation deproteinization; **4**: deproteinized fSSO; **5**: fermented fSSO (F-fSSO); **6**: fSSO.

The solid residue obtained after fermentation was converted to chitin after demineralization by strong acid (2N HCl) then subjected to subsequent analysis. Fourier-transform infrared spectroscopy (FTIR) was used to analyze the quality of prepared chitins and their intermediate products by using commercial chitin (Sigma, St. Louis, MO, USA) as a control. As shown in Figure 6, the prepared chitin was found to be an α-chitin based on the OH and NH stretching vibration at 3443 and 3267 cm^−1^, symmetric stretching vibration of CH, CH_3_ and the asymmetric stretching vibration of CH_2_ at 2891 to 2962 cm^−1^, deformation of CH_3_ and the wagging of CH (Amide III) at 1380 and 1313 cm^-1^, and asymmetric oxo bridge and C-O stretching vibration at 1030 to 1157 cm^−1^ [41,42]. Finally, the peaks at 1662, 1626, and 1558 cm^−1^ represented amide I and amide II, respectively [1,43]. The chitins prepared by chemical method and fermentation method were almost similar to the commercially available chitin, indicating that deproteinization by TKU051 fermentation did not significantly change the structure of chitin. Therefore, chitin extraction from fSSO by *P. elgii* TKU051 could be feasible. 

### 2.6. Dyes Adsorption Ability of Chitins 

**Figure 7:**
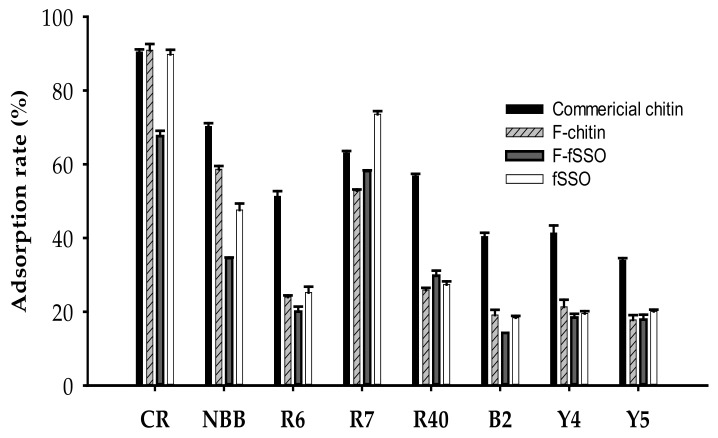
The rate of dye adsorption by chitins, fermented fSSO (F-fSSO), and fSSO. CR, Congo Red; NBB, Naphthol blue-black; R6, Red No. 6; R7, Red No. 7; R40, Red No. 40; B2, Blue No. 2; Y4, Yellow No. 4; Y5, Yellow No. 5. The error bars are the standard deviation of three replicates.

Dyes are used by different industries to color their products, but the limited fixation capacity often results in the generation of a large amount of dye wastewater that is discharged into the river and causes huge problems of pollution [44,45]. Common treatment methods, including radiation, ion exchange, filtration, chemical destruction, are categorized into physical methods, chemical methods, and biological methods according to their properties [46]. Nevertheless, these methods are usually expensive. Therefore, at present, the adsorption method is primarily used to remove the dye in the water body. It has been reported that seafood processing by-products can be used as effective adsorbents after some modification [47,48,49]. As shown in Figure 7, although the raw material fSSO showed some adsorption effect on Congo Red, naphthol blue-black, and red no. 7, the reclamation of the containing proteins and prevention of the protein caused secondary pollution during the adsorption process. Therefore, the fSSO deproteinized by *P. elgii* TKU051 fermentation and then demineralized by acid (F-chitin) were also carried out to estimate the dye adsorption effect compared with that of commercial chitin. The results showed that the effect of F-chitin was comparable to that of commercial chitin on the adsorption against Congo Red (90.48 and 90.91%, respectively). Considering the other dyes, commercial chitin expressed a higher adsorption capacity than F-chitin and fermented fSSO (F-fSSO). Several reports have shown that chitin has a high adsorption capability on Congo Red [19]. The dye adsorption capacity of chitin may be related to its porous structure, functional groups, and the degree of deacetylation [50,51]. In this study, by showing a comparable Congo Red adsorption rate, the chitin from F-fSSO may hold great potential in removing this dye from wastewater.

## 3. Materials and Methods

### 3.1. Materials 

Squid pens and shrimp processing wastes (shrimp heads, shrimp shells) were obtained from Shin-Ma Frozen Food Co. (I-Lan, Taiwan) and Sunmake Enterprise Co. (Taipei, Taiwan). Cooked shrimp heads and cooked shrimp shells were obtained by treating shrimp wastes at 100 °C for 20 min, and then drying in an oven (at 70 °C for 48 h), or by lyophilization, respectively. Azocasein, β-ME, PMSF, Congo Red, and naphthol blue-black were purchased from Sigma-Aldrich Co. (St. Louis, MO, USA). Red no.6, Red no.7, Red no.40, Blue no.2, Yellow no.4, and Yellow no.5 were purchased from First Chemical Works Co. (Taipei, Taiwan). All other reagents used in this study were of the highest grade available.

### 3.2. Isolation and Identification of Protease-Producing Bacteria 

The strain was isolated from the soil of Tamkang University (New Taipei, Taiwan). The soil samples were serially diluted with 0.9% saline and spread on SPP-containing medium (1% SPP, 0.1% K_2_HPO_4_, 0.05% MgSO_4_·7H_2_O, *w/v*) with 1.5% (*w/v*) agar, and incubated for 48 h [21]. The single colonies were combined with 100 mL of SPP-containing medium then incubated for 72 h. The culture supernatant was collected for the analysis of protease activity. The strain with the highest protease activity was identified through morphological, physiological, and biochemical properties, as well as 16s rRNA sequencing.

### 3.3. Measurement of Protease Activity 

Fifty μL of a 1% (*w/v*) azocasein solution were added to 50 μL of cell-free supernatant (containing protease) and the mixture was immediately incubated at 37 °C for 30 min. The reaction was stopped by mixing with 300 μL of 5% (*w/v*) trichloroacetic acid (TCA, Katayama Chemical, Osaka, Japan). Finally, the sample was centrifuged at 13,000 rpm for 10 min and 150 µL of the supernatant was mixed with an equal amount of 0.5 N NaOH. The absorbance of the mixture was measured at 450 nm using a Microplate Absorbance Reader (BIO-RAD, Hercules, CA, USA). One unit was defined as an increase of A_450 nm_ of 0.01 after incubation for 1 min [52,53,54].

### 3.4. Gel Electrophoresis 

Zymography was performed on a 12.5% resolving gel containing 0.05% gelatin. After electrophoresis, the gels were rinsed in 5% Triton X-100 for 1 h at room temperature and rinsed in 50 mM Tris-HCl buffer (pH 7) for 1 h. The gels were then rinsed in 50 mM Tris-HCl buffer (pH 7) and incubated at 37 °C for 1 day. The gels were stained with Coomassie Brilliant Blue R-250 (CBR, Sigma, St. Louis, MO, USA).

### 3.5. Effect of Temperature and pH on Protease Activity and Stability

The optimal temperature of the protease was assessed by incubating the enzyme and substrate mixture at different temperatures (30–100 °C) for 30 min. The thermal stability was determined through the residual activity after pretreating the enzyme under different temperatures for 30 min. The optimal pH was determined using a range of buffers including citrate acetate (pH 3–6), Tris-HCl (pH 7–9), Na_2_CO_3_-NaHCO_3_ (pH 9–11), Na_2_PO_4_-NaOH (pH 12). The pH stability was investigated by pretreating the enzyme at different pH and measuring the residual activity at pH 7.

### 3.6. Effect of Various Chemicals on the Activity of Protease 

The enzyme was pretreated with various metal ion salts, β-ME, inhibitor (PMSF and EDTA from Sigma, St. Louis, MO, USA), and surfactants (Tween 20, Tween 40, and Triton X-100, from Merck, Darmstadt, Germany) for 30 min at room temperature. After that, the substrate was added to the mixture and the residual protease activity was tested.

### 3.7. Substrate Specificity

Various substrates were used to investigate the substrate specificity of *P. elgii* TKU051 protease, including albumin, hemoglobin, keratin, myoglobin, elastin, gelatin, fibrin, casein, SSP, SHP, SMP, FHP, and SBP. Casein was used as a control for determining substrate specificity. Protease assay and substrate specificity were determined following the Todd method [29].

### 3.8. 2,2-Diphenyl-1-Picrylhydrazyl (DPPH) Radical Scavenging Activity 

The DPPH radical scavenging activity analysis was as per the method of Doan et al. [55].

### 3.9. α-Glucosidase Inhibitory Activity

α-glucosidase inhibitory activity was analyzed following the method of Tran et al. [56].

### 3.10. Fermentation Conditions

*P elgii* TKU051 was grown in a 250 mL flask containing 100 mL of the liquid medium (1% each kind of shrimp waste, 0.1% K_2_HPO_4_, 0.05% MgSO_4_·7H_2_O, *w/v*) at 37 °C, and 150 rpm for 7 days. The nonfermentation group was prepared in similar conditions (mentioned above) with the exception that the bacterial strain was not inoculated in the medium. The shrimp wastes were fSHO, fSHL, fSSO, fSSL, cSHO, cSHL, cSSO, and cSSL.

### 3.11. Deproteinization Assay 

Deproteinization of shrimp waste was examined through the fermentation method and the alkali method. In the fermentation method, 1% of TKU051 seed culture was added into basal medium containing 1% (*w/v*) of shrimp waste and incubated at 37 °C and 150 rpm for a week. In the alkali method, 1 g of sample was mixed with 50 mL of 5% NaOH (*w/v*). The mixture was allowed to react at 80 °C for 4 h then centrifuged at 1000 rpm for 10 min. The supernatant was then used to analyze the protein concentration by the Lowry method [57,58]. The rate of deproteinization was calculated by following [59]:

(1) $$\mathrm{Deproteinization}\;\mathrm{rate}\;\;\left(\%\right)=\;\frac{\left[\left(P_{o}\times O\right)-\left(P_{R}\times R\right)\right]}{\left(P_{o}\times O\right)}\times 100\%$$

where *P_o_* and *P_R_* are protein content (%) before and after deproteinization while *O* and *R* are the dry weight of the sample before and after deproteinization.

### 3.12. Chitin Extraction 

The solid residue obtained after deproteinization was washed with water and dried in an oven at 70 °C. One gram of sample was mixed with 10 mL of 2N HCl at room temperature for 1 h. The precipitate was washed with water until neutral pH was obtained.

### 3.13. FTIR Analysis 

Chitins and their mid-products recovered from shrimp waste were analyzed by FTIR spectrometer (Tensor 27, Bruker, Ettlingen, Germany) for characterization. Spectra were collected from 4000 to 400 cm^−1^ following the KBr method.

### 3.14. Dye Adsorption 

Ten milligrams of adsorbent were mixed with 5 mL of dye solution (0.002%, *w/v*) in a centrifugation tube then placed on a rotary shaker at 150 rpm for 1 h. The mixture was centrifugated at 6000 rpm for 10 min. The absorbance of the mixture was measured by an ELISA reader (Epoch, Bio Teck, Winooski, VT, USA). The maximum absorption wavelengths of Congo Red, Naphthol blue-black, Red No. 6, Red No. 7, Red No. 40, Blue no.2, Yellow No. 4, and Yellow No. 5 were 490, 620, 510, 530, 550, 610, 430, and 480 nm, respectively. The adsorption rate was calculated using the following formula:

(2) $$Adsorption\;rate\;\left(\%\right)=\left[\left(C-S\right)/C\right]\times 100\%$$

where *C* is absorbance before adsorption and *S* is absorbance after adsorption.

## 4. Conclusions

Chitinous fishery by-products are available in large quantities and are low in price. The use of these materials for enzyme and chitin production via a microbial conversion method could increase their economic value and also help in preventing pollution caused by the acid/alkali used for demineralization/deproteinization. In this study, the production of the proteases of *P. elgii* TKU051 on an SPP-containing medium was explored by gelatin zymography analysis. Following that, four proteases of MWs 100, 57, 43 and 34 kDa were detected in the culture supernatant of *P. elgii* TKU051. Additionally, the hydrolysates prepared from *P. elgii* TKU051 crude enzyme cocktail possessed antioxidant and α-glucosidase inhibitory activities, and the deproteinization capacity of *P. elgii* TKU051 on different kinds of shrimp waste exhibited a high value (over 94%). The chitin prepared from the deproteinization of fSSO using *P. elgii* TKU051 was characterized by FTIR analysis and revealed a pattern similar to that of high-grade commercially available chitin. Finally, the obtained chitin exhibited potential dye adsorption capacity on Congo Red. Hence, *P. elgii* TKU051 could be an efficient candidate for the conversion process of chitinous fishery by-products to produce proteases and chitin.

## Figures and Tables

**Table 1:** A summary of proteases produced by *Paenibacillus* strains.

Strain	MW (kDa)	C/N Source	Ref.
*P. elgii* TKU051	100, 57, 43, 34	SPP	This study
*P. woosongenisis* TKB2		chicken feather	[28]
*P. mucilaginosus* TKU032	32	shrimp head powder	[19]
*Paenibacillus* sp. TKU047	32	shrimp head powder	[22]
*Paenibacillus* sp. TKU042	35	SPP	[29]
*Paenibacillus* spp. BD3526	35	wheat bran	[30]
*P. polymyxa* EJS-3	63.3	tryptone, beef extract	[31]
*P. larvae*	87, 74, 42, 40	MYPGP broth	[25]
*Paenibacillus* sp. TKU052	31 ^1^	demineralized crab shell powder	[21]
*P. lautus* CHN26	53.92 ^2^		[32]
*P. tezpurensis* sp. now. AS-S24-II	43	casein and ammonium sulphate	[33]
*P. polymyxa* SCE2 and *P. peoriae* NRRL BD-62	210, 50, 35, 20	thiamine/biotin/nitrogen broth	[26]

^1^ Two proteases with a similar MW; ^2^ calculated MW (in kDa).

**Table 2:** Effect of metal ions, inhibitors, surfactants and reducing agents on the protease activity of *P. elgii* TKU051 crude enzyme cocktail.

Chemical	Concentration	Relative Activity (%)
None		100.00 ± 1.54
MgSO_4_	5 mM	113.34 ± 1.15
CuSO _4_	5 mM	NA
FeSO_4_	5 mM	87.24 ± 2.58
CaCl_2_	5 mM	108.12 ± 2.80
ZnSO_4_	5 mM	NA
MnSO_4_	5 mM	364.04 ± 7.50
ZnCl_2_	5 mM	NA
BaCl_2_	5 mM	99.07 ± 2.28
PMSF	5 mM	52.81 ± 8.18
EDTA	5 mM	20.16 ± 0.76
SDS	2 mM	22.50 ± 2.65
*β*-ME	2 mM	88.91 ± 3.93
Tween 20	2%	177.26 ± 0.44
Tween 40	2%	204.59 ± 5.14
Triton X-100	2%	127.96 ± 7.62

Values are mean ± standard deviation of three experiments. NA, no activity.

**Table 3:** Substrate specificity of protease produced by *P. elgii* TKU051 crude enzyme cocktail.

Substrate	Relative Activity (%)
Casein	100.00 ± 0.19
Hemoglobin	40.80 ± 0.16
Albumin	36.28 ± 0.41
Myoglobin	31.80 ± 0.20
Elastin	25.80 ± 0.34
Fibrin	29.07 ± 0.44
Gelatin	23.54 ± 0.62
Keratin	58.85 ± 0.15
SSP	28.49 ± 0.24
SHP	24.01 ± 0.46
FMP	34.96 ± 0.29
SMP	44.50 ± 0.93
FHP	31.99 ± 0.78
SBP	29.81 ± 0.22

Values are mean ± standard deviation of three experiments.

**Table 4:** Deproteinization of different kinds of shrimp wastes.

Kind of Shrimp Waste	Deproteinization Rate (%)		Residual Protein Content (%)	
	Fermentation	Nonfermentation	Fermentation	Nonfermentation
cSHL	95.90 ± 0.18	50.29 ± 1.71	1.99 ± 0.13	24.17 ± 0.47
cSHO	94.47 ± 0.66	42.38 ± 3.54	2.68 ± 0.31	27.91 ± 1.09
cSSL	94.61 ± 0.29	38.00 ± 0.45	2.25 ± 0.12	25.86 ± 0.10
cSSO	94.40 ± 0.24	43.09 ± 4.89	2.37 ± 0.07	24.03 ± 2.17
fSHL	96.86 ± 0.33	55.43 ± 1.11	1.51 ± 0.19	21.45 ± 0.57
fSHO	95.16 ± 0.26	53.25 ± 3.97	2.22 ± 0.15	21.43 ± 1.99
fSSL	95.23 ± 0.14	50.59 ± 3.41	2.10 ± 0.04	21.53 ± 2.04
fSSO	94.36 ± 0.19	46.74 ± 2.59	2.46 ± 0.01	23.67 ± 2.23

Values are mean ± standard deviation of three experiments.

**Table 5:** Deproteinization of different amounts of fSSO by *P. elgii* TKU051 protease.

Amount of fSSO	Deproteinization Rate (%)		Residual Protein Content (%)	
	Fermentation	Nonfermentation	Fermentation	Nonfermentation
1%	97.45 ± 0.03	51.48 ± 1.45	1.11 ± 0.04	21.15 ± 0.70
2%	46.93 ± 4.18	50.21 ± 2.16	23.12 ± 1.96	21.69 ± 0.35
3%	50.60 ± 3.32	42.94 ± 0.57	21.51 ± 1.07	24.89 ± 0.73
4%	48.81 ± 3.66	16.26 ± 0.30	22.31 ± 1.78	36.54 ± 1.47
5%	33.34 ± 0.06	8.85 ± 3.66	29.08 ± 1.12	39.72 ± 0.55

Values are mean ± standard deviation of three experiments.
